# Nuclear lamin A/C harnesses the perinuclear apical actin cables to protect nuclear morphology

**DOI:** 10.1038/s41467-017-02217-5

**Published:** 2017-12-14

**Authors:** Jeong-Ki Kim, Arghavan Louhghalam, Geonhui Lee, Benjamin W. Schafer, Denis Wirtz, Dong-Hwee Kim

**Affiliations:** 1 KU-KIST Graduate School of Converging Science and Technology, Korea University, Seoul, 02841 South Korea; 2 Department of Civil and Environmental Engineering, University of Massachusetts Dartmouth, Dartmouth, MA 02747 USA; 3 Institute for NanoBioTechnology, The Johns Hopkins University, Baltimore, MD 21218 USA; 4 Department of Civil Engineering, The John Hopkins University, Baltimore, MD 21218 USA; 5 Department of Chemical and Biomolecular Engineering, The John Hopkins University, Baltimore, MD 21218 USA; 6 Johns Hopkins Physical Sciences—Oncology Center, The Johns Hopkins University, Baltimore, MD 21218 USA; 7 Departments of Pathology and Oncology and Sydney Kimmel Comprehensive Cancer Center, The Johns Hopkins School of Medicine, Baltimore, MD 21205 USA

## Abstract

The distinct spatial architecture of the apical actin cables (or actin cap) facilitates rapid biophysical signaling between extracellular mechanical stimuli and intracellular responses, including nuclear shaping, cytoskeletal remodeling, and the mechanotransduction of external forces into biochemical signals. These functions are abrogated in lamin A/C-deficient mouse embryonic fibroblasts that recapitulate the defective nuclear organization of laminopathies, featuring disruption of the actin cap. However, how nuclear lamin A/C mediates the ability of the actin cap to regulate nuclear morphology remains unclear. Here, we show that lamin A/C expressing cells can form an actin cap to resist nuclear deformation in response to physiological mechanical stresses. This study reveals how the nuclear lamin A/C-mediated formation of the perinuclear apical actin cables protects the nuclear structural integrity from extracellular physical disturbances. Our findings highlight the role of the physical interactions between the cytoskeletal network and the nucleus in cellular mechanical homeostasis.

## Abstract (web summary)

An actin cap protects the morphology of the nucleus during cellular mechanical stress. Here, the authors show that the nuclear lamina protein lamin A/C mediates the formation of the actin cap in response to stress, and model the distribution of forces in the presence and absence of the actin cap.

## Introduction

Ill-shaped nuclear morphology is a hallmark of laminopathies, which are relatively rare genetic diseases including progeria syndrome^1^, congenital muscular dystrophy^2^, dilated cardiomyopathy^3^, restrictive dermopathy^4^, and familial partial lipodystrophy^5^. Laminopathic diseases are mainly attributed to mutations in the lamin A/C gene (*LMNA*) that encodes for the filamentous lamin A/C proteins, a major component of the nuclear lamina^6,7^. Recent micromechanical measurements have shown that, similar to highly metastatic tumor cells that typically display a higher mechanical compliance than non-metastatic cells^8,9^, cells that feature mutant *LMNA* have a low stiffness compared to *LMNA*-present cells^10,11^, illustrating how lamin A/C-mediated nuclear mechanics is correlated with the physical properties of the cell.

*LMNA*-mutant cells and cells deficient in lamin A/C lack the perinuclear apical actin cables (or more simply, actin cap)^12,13^ that are present on top of the interphase nucleus in a wide range of adherent somatic cells, including fibroblasts and endothelial cells^14^. The actin cap is composed of actomyosin filament bundles and connected to the nuclear envelope through the linkers of nucleoskeleton and cytoskeleton (LINC) molecular complexes consisting of nuclear membrane-embedded nesprin isoforms and Sad1-UNC84 Homology (SUN)-domain proteins that are anchored to the nuclear lamina^14,15^.

Actin stress fibers of the actin cap are also terminated by particularly large focal adhesions at the extreme periphery of the cell^16^. Owing to the additional mechanical tension provided by the unique topology of the actin cap^17,18^, enlarged actin cap-associated focal adhesions have a major role in mechanosensation, the ability of cells to sense the mechanical compliance of their local environment^14,19^. Moreover, as systematically assessed from the micro-contact-printed fibronectin patterning assay, the morphology of the interphase nucleus of the adherent cells is also tightly regulated by the actin cap^13^. Therefore, the selective removal of the actin cap by the treatment with a low dose of actin depolymerizing drug^16,20^, the molecular disruption of LINC complexes^9,21^, or the elevation of substrate compliance^21,22^ results in the profound deformation of nuclear morphology.

Previously, we showed that the spatial remodeling of lamin A/C and the formation of an actin cap are closely related^18^. Owing to this mechanical interaction, biophysical signals are transmitted from the cytoskeleton to intranuclear chromosomal organizations^18^. This explains the differential reshaping of the nuclear morphology in response to substrate compliance that regulates the cytoskeletal tension^16^ and gradual reduction of nuclear volume during cell detachment from the substrate, where morphological transition from a smooth surface to a folded surface is typically involved due to the loss of the compressive force of the actin cap^23^.

To further delineate the role of the actin cap in regulating the nuclear morphology of mechanically stressed cells, we incorporated cellular responses to the substrate deformation with the computational models developed in the present work. Substrate stretching is a well-designed tool used to apply tensile force to the cell, which enables the monitoring of cellular mechano-responses, such as the reorganization of the cytoskeletal structure^24^ and realignment of the cell body^25^. Moreover, as direct experimentation alone can help to address the impact of mechanical deformations on cellular behaviors, it does not provide a way of mapping or interpreting the force distribution within the cell. Therefore, continuum models implemented in a finite element framework have been developed to better understand cell mechanics by modeling entire cells^26–29^. This approach was able to successfully capture the essential nonlinearities between the cell structure and responses^28^, and has provided new insight into the close relationship between extracellular mechanical stimuli and intracellular responses.

An accurate mechanical model of a cell can provide the intracellular stress and strain fields that are associated with direct mechanical stimulation and associated response. Various phenomena in cell behavior can then be separated from those explained by the basic mechanical response. This separation is vital in determining if a cellular response is regulated by the mechanotransduction circuitry of the cell or simply by a direct physical response to external stimuli. The key feature of the model pursued herein is the discrete embedment of the recently identified perinuclear apical actin cables, i.e. actin cap in the cytoskeletal network.

The architecture of the actin based cytoskeletal network has long been implicated to mediate cellular functions^30,31^. We investigated the effect of substrate stretching on the nuclear morphology of mouse embryonic fibroblasts in the presence and absence of lamin A/C. Our study combines experimental results and computational simulation to directly demonstrate a nuclear defense mechanism in a physically stressed cell, which further reveals the underlying load-carrying mechanism of the cell. From this work, we show that cellular mechanotransduction is attenuated in laminopathic cells and that the lamin A/C-mediated formation of the actin cap is necessary to maintain the structural integrity of the nucleus in response to external physical stimuli.

## Results

### Substrate stretching changes nuclear morphology

**Fig. 1:**
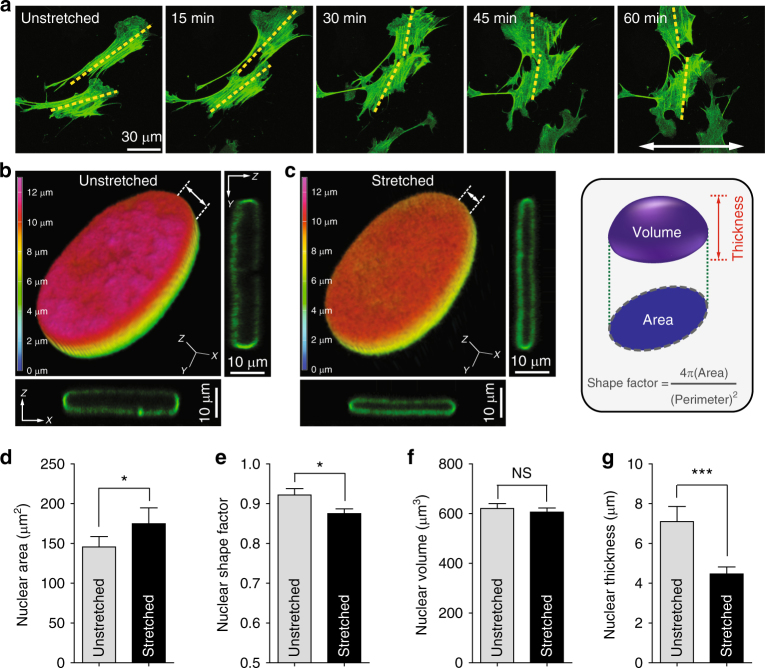
Stretch-induced nuclear morphological changes in mouse embryonic fibroblasts. **a** Cell re-orientation under uniaxial cyclic stretching of the substrate. Randomly seeded EGFP–LifeAct-transfected mouse embryonic fibroblasts (MEFs) onto the stretchable PDMS thin film were re-oriented in response to the substrate stretching (8% strain ratio and 1 Hz frequency) by the reorganization of actin cytoskeleton perpendicular to the substrate stretching direction (indicated by white arrow). Yellow dashed lines indicate the main axis of the actin cytoskeleton traversing the cell body. Refer to Supplementary Figure 1 for the verification of experimental setup. **b**,** c** Stretch-induced nuclear flattening. A GFP–lamin-A-transfected MEF was imaged under the same stretching condition for 1 h. Pseudo-colored *z*-depth rendering and *xz* and *yz* cross-sectional views of the three-dimensionally reconstructed nuclei represent stretch-induced nuclear flattening. **d**–**g** Quantification of nuclear morphological changes. Footprint size (i.e., area) and shape factor estimated from a projected nuclear morphology onto the *XY*-plane depict a slight enlargement and elongation of the stretched nuclei (**d**, **e**). Nuclear volume and thickness (i.e., the maximum length from the basal surface to the apical surface) of the three-dimensionally reconstructed nuclei reveal that substrate stretching generates a pressing force vertical to the nucleus of adherent cells, whereas nuclear volume was conserved (**f**, **g**). To avoid out-of-focus images and to prevent photobleaching of transfected cells, each image was captured every 15 min for ~1 h (**a**). Overall, >30 cells were examined per condition (**d**–**g**), where error bars indicate S.E.M. and statistical differences were calculated using the unpaired *t*-test, ***p < 0.0001, *p < 0.01, NS not significant (*p* > 0.05)

To verify that our custom-made vacuum-controlled cyclic substrate stretcher reliably transmitted mechanical stimuli to the cell, we first quantified the cell re-orientation induced by 1 Hz, 8% uniaxial cyclic stretching of the deformable polydimethylsiloxane (PDMS) thin film (refer to Methods, Supplementary Note 1, and Supplementary Figure 1). These frequency and strain values are within the physiological range for various cell types such as pulmonary and alveolar endothelial cells, cardiac myocytes, and fibroblasts^32–35^. Although mouse embryonic fibroblasts (MEFs) adherent to unstretched static PDMS films were randomly oriented, 1 h of substrate stretching induced significant cell re-orientation (Fig. 1a; Supplementary Figure 1D, E; Supplementary Movie 1). The extent of cell orientation, defined as the angle between the longest chord through the cell and the stretching direction, significantly increased from ~45° (corresponding to random orientation) to ~60° (Supplementary Figure 1F–H). This is consistent with previous experimental and theoretical results showing that cells are re-aligned away from the direction of the substrate stretching^25,36^. In our experimental setup, no significant change of cell population was detected and time-lapse monitoring of the EGFP–LifeAct-transfected MEFs confirms that actin cytoskeletal remodeling was accompanied by substrate stretching (Fig. 1a). These results further demonstrated that stretch-induced cell re-orientation in our substrate stretching platform was not a trivial result of cell detachment and re-attachment, but was the result of mechanical stimulation from the basal surface of the cell properly transmitted into the cytoskeleton.

Extracellular forces are transferred from the underlying substrate to intracellular organelles via integrins in focal-adhesion protein complexes that anchor the actin stress fiber^37–39^. These stress fibers then transmit mechanical signals to the lamin-bound nucleus^18,40^. Therefore, we examined the three-dimensionally (3D) reconstructed nuclei of GFP-lamin A-transfected MEFs to determine whether the nuclear morphology was also affected by substrate stretching (Fig. 1b–g). Although the nuclear volume remained unchanged, surprisingly, we found that nuclear thickness was significantly reduced after substrate stretching (Fig. 1f, g). Computer-aided 3D surface rendering of the nucleus clearly delineated this reduction in height (see depth-dependent color codes, Fig. 1b, c). As nuclear morphology projected to the *XY*-plane showed an enlarged nuclear area (Fig. 1d) and an elongated nuclear shape (Fig. 1e), we concluded that the lateral deformation of the underlying substrate generates a pressing force on the nucleus perpendicular to the adherent cell, where nuclear volume was conserved. Moreover, we noted that the nuclear morphometric change in the vertical axis (i.e., nuclear thickness) was larger than the changes in the 2D-projected nuclear morphology (i.e., nuclear area and shape factor). Therefore, nuclear flattening is a more important morphometric change (representing the stretch-induced nuclear deformation) than the lateral expansion or reduction of the nucleus.

Together, these results reveal that the extracellular mechanical stresses generated from the cyclic stretching of the substrate modulate not only the global cell orientation by reorganizing the actin cytoskeleton, but also the 3D-nuclear morphology by applying vertical pressuring forces on the nucleus.

### Nuclear lamin A/C determines 3D-nuclear morphology

Stretch-induced reorganization of the actin cytoskeleton and 3D-nuclear deformation suggest that nucleus-cytoskeletal connection could couple extracellular physical disturbances and nuclear morphology. In particular, mesh-structured nuclear lamin A/C, a partner for nucleo-cytoskeletal molecular connections in the nuclear envelope^22,41^, is well known to modulate nuclear shape via remodeling of actin cytoskeleton in a two-dimensional cell culture^22,42,43^. Therefore, we investigated the intrinsic effect of lamin A/C on 3D-nuclear morphology by comparing lamin A/C-bearing wild-type (WT) MEFs and lamin A/C knockout MEFs (*Lmna*^−/−^) placed in cell culture dishes to see if nuclear lamin A/C is involved in stretch-induced nuclear deformation.

**Fig. 2:**
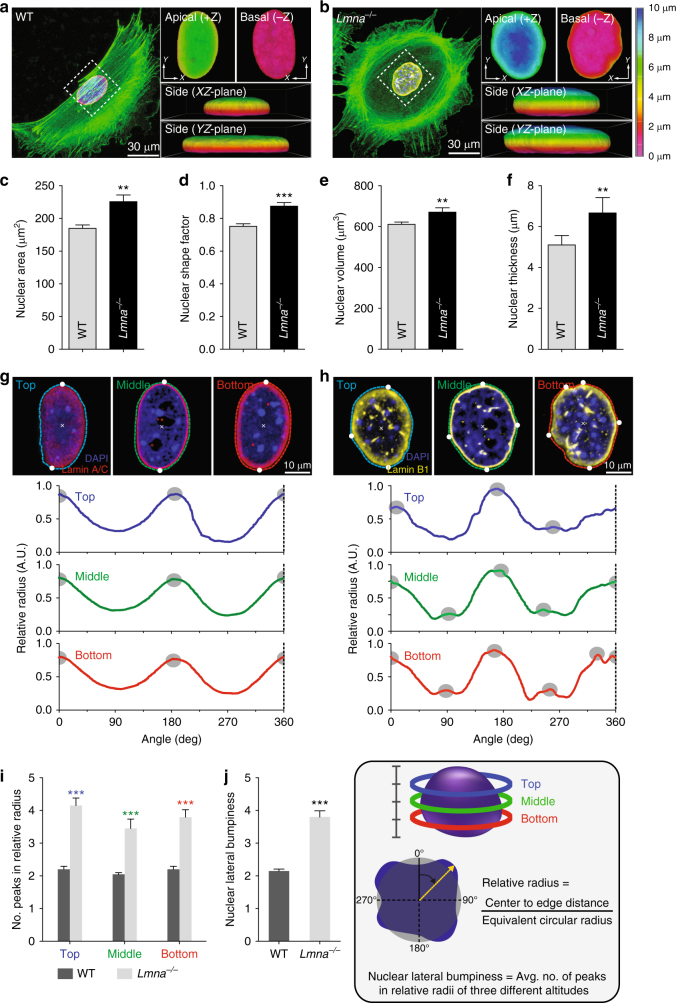
Effect of nuclear lamin A/C on nuclear morphology. **a**–**f** Lamin A/C-dependent difference of nuclear morphology in MEFs placed in a glass bottom dish. Representative cell and nuclear morphology in lamin A/C-present control wild-type (WT) and lamin A/C-deficient *Lmna* knockout (*Lmna*^−/−^) MEFs were captured by 3D reconstruction of immunofluorescence confocal images along the *z*-axis (**a**, **b**). Note that apical, basal, and side views of pseudo-colored 3D *z*-depth rendered nuclei display the differences in the nuclear surface textures. *Lmna*^−/−^ cells displayed more spread (**c**), rounder (**d**), larger (**e**), and thicker (**f**) nuclei than the WT control cells. **g**–**j** Comparison of lamin A/C-dependent lateral deformation of the nucleus. Tracking surface contours in three different altitudes in a nucleus displays significantly more peak positions (gray dots) in *Lmna*^−/−^ cells than WT cells (**g**–**i**), representing more protrusions on the nuclear surface of the *Lmna*^−/−^ MEFs. Relative radius is the ratio between the nuclear center-to-edge distance and the equivalent circular radius, where top (blue), middle (green), and bottom (red) parts correspond to the top 25%, top 50%, and top 75% positions along the *z*-axis of the nucleus, respectively. Average number of peaks in relative radii of three different altitudes was termed by the nuclear lateral bumpiness (**j**). In **c**–**j**, >30 cells were analyzed per condition. Error bars represent S.E.M. of averaged values and statistical differences were calculated using the unpaired *t*-test; ***p < 0.0001, **p < 0.001

To quantitatively measure the 3D morphology of the nucleus, we analyzed 3D-reconstructed confocal images of the nucleus (Fig. 2). Depth-dependent color-coded 3D-rendering of the nuclear surface distinguished the dilated nuclear morphology in *Lmna*^−/−^ MEFs from a vertically compressed nuclear shape in WT MEFs (Fig. 2a, b). The projection of the 3D-reconstructed nucleus to the *XY*-plane showed a more enlarged and rounded nuclear shape in *Lmna*^−^^/−^ MEFs than in WT MEFs (Fig. 2c, d), which is consistent with our previous results obtained from the high throughput cellular phenotyping (htCP) analysis performed using a 2D cell culture^13^. Consequently, *Lmna*^−/−^ MEFs displayed a larger nuclear volume and thickness (Fig. 2e, f).

Moreover, as we noted that *Lmna*^−/−^ cells typically displayed non-smooth protrusions along the outer contour of the nuclei, we quantified the degree of unevenness of the nuclear surface, depending on nuclear lamin A/C. (Refer to apical (+*z*) and basal (−*z*) views of pseudo-colored *z*-depth rendered nuclei, Fig. 2a, b). The relative radii of the nucleus were tracked in three different altitudes of the nucleus, each representing the top, middle, and bottom portion of the nucleus (Fig. 2g–i). Compared to the nucleus of the control WT MEFs showing only two peak values in the relative radius, where each peak corresponds to the maximum circular radius along the nuclear surface (Fig. 2g, i), the *Lmna*^−/−^ MEFs displayed more than two peaks in different altitudes, demonstrating the irregular nuclear morphology (Fig. 2h, i).

Therefore, nuclear lateral bumpiness, defined as the average number of peaks along the relative radii in three different altitudes, was significantly enhanced in the case where the nuclear lamin A/C was deficient in the cell (Fig. 2j). This result supports pathological evidence showing that cells in progeria patients where *LMNA* is mutated display an irregular nuclear shape, uncontrolled nuclear sizing, and weak mechanical integrity due to the loss of lamin A/C-mediated nucleus-cytoskeletal connectivity^22,44^.

Together, topological characterization of the nuclei of lamin A/C presenting WT MEFs and lamin A/C lost *Lmna*^−/−^ MEFs reveals that lamin A/C serves as the primary determinant of 3D-nuclear morphology.

### Lamin A/C-mediated actin cap prevents nuclear deformation

The perinuclear apical actin cables, collectively termed actin cap, are the specialized actin stress fibers composed of parallel-aligned bundles of actomyosin filaments identified on top of the nucleus^14^. Recent studies have unveiled various topology-dependent functions of the actin cap in mediating nuclear mechanics^13,14,16,45^. For example, the actin cap controlled cell–nucleus coupled migration through nuclear envelop (NE)-embedded LINC proteins^13^ and cytoskeletal tension mainly applied by the actin cap remodeled the spatial organization of nuclear lamin A/C^18^. Therefore, we asked whether stretch-induced extracellular mechanical stimuli triggered the formation of the actin cap that could reform the nuclear morphology and whether this mechano-response was regulated by lamin A/C.

**Fig. 3:**
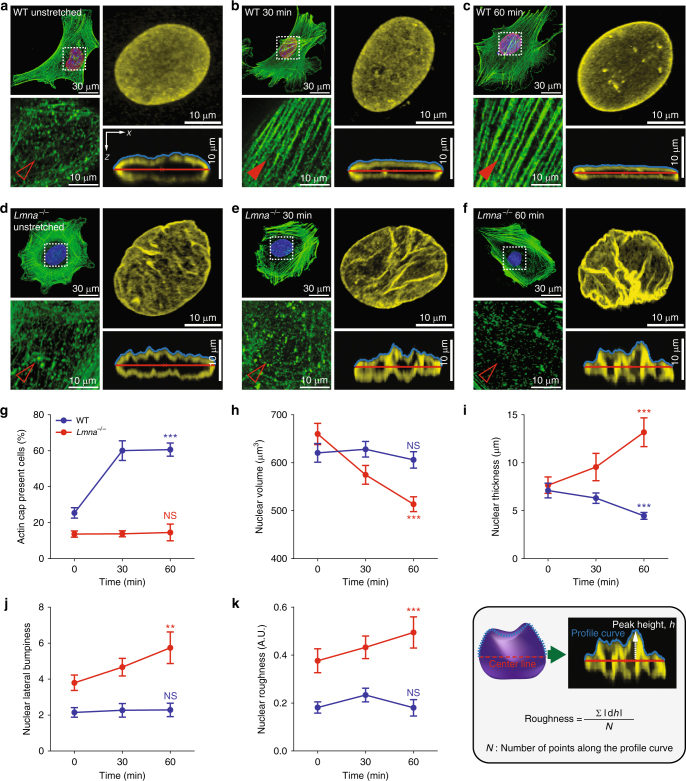
Lamin A/C-dependent differential formation of an actin cap and nuclear deformation in response to substrate stretching. **a**–**f** Representative F-actin organization and nuclear morphology of lamin A/C-present WT (**a**–**c**) and lamin A/C-deficient *Lmna*^−/−^ (**d**–**f**) MEFs at different time points of the substrate stretching (0, 30, 60 min). Insets display details of F-actin organization in the apical region of the nucleus. Full and empty arrowheads indicate the presence and absence of the perinuclear actin cap, respectively. Nuclear morphology of lamin B1-stained nuclei (yellow) indicates distinct evolution of 3D-nuclear shape in response to substrate stretching, where maximum intensity projection onto the *XY*-plane was performed using upper hemispheres of the 3D-reconstructed nuclei to highlight the detailed nuclear surface texture. The cross-sectional side view was captured along the *XZ*-plane crossing the center of the nucleus. **g**–**k** Stretch-dependent formation of an actin cap (**g**) and changes of nuclear volume (**h**), thickness (**i**), lateral bumpiness (**j**), and surface roughness (**k**) in WT (blue) and *Lmna*^−/−^ (red) MEFs. Note that nuclear flattening with volume conversation (**h**, **i**) and nuclear surface roughening with volume reduction (**j**, **k**) are the stretch-induced characteristic features of the actin cap forming WT and actin cap non-forming *Lmna*^−/−^ cells (**g**). In **i** and **k**, nuclear thickness was defined as the maximum height in the vertically cross-sectioned image of the 3D-reconstructed lamin B1-stained nucleus and the height variation along the apical surface was termed as surface roughness. In **g**, >150 cells were examined per condition and in **h**–**k**, >30 lamin B1-stained nuclei were analyzed per condition. Error bars represent the S.E.M. of averaged values. Unpaired *t*-test was applied to compare unstretched control cells (0 min) and fully stretched cells (60 min). ***p < 0.0001, **p < 0.001, NS not significant (*p* > 0.05)

To systematically answer these questions, we first quantified the fraction of cells that formed an actin cap by gradually increasing the stretching time (Fig. 3a–c). Consistent with previous results which showed that the actin cap was selectively disrupted in cells placed on the compliant hydrogels^16^, MEFs placed on the deformable PDMS films did not form an actin cap (Supplementary Figure 2A), whereas they maintained their organized basal actin stress fibers (Fig. 3a). However, the fraction of cells forming an organized actin cap rapidly increased when subject to cyclic stretching of the underling membranes without significantly affecting the basal actin structure (Fig. 3a–c; Supplementary Figure 2A–C). In contrast, lamin A/C absent *Lmna*^−/−^ MEFs did not form an actin cap in response to the same mechanical stimulation (Fig. 3d–g; Supplementary Figure 2D–F). As the organization of basal actin stress fibers was not affected in both the control WT and *Lmna*^−/−^ cells (Fig. 3a–f; Supplementary Figure 2A–F), these results strongly suggest that the formation of an actin cap is a distinct phenotypic feature of stretch-induced cellular mechanotransduction and lamin A/C is a biophysical transducer regulating mechanical signal pathways ranging from the extracellular force to the intracellular nuclear reshaping.

Therefore, together with the results that demonstrate that *Lmna*^−/−^ MEFs intrinsically displayed more deformed nuclear shape than the WT cells (Fig. 2) and they showed a low reactivity to the stretch-induced formation of the actin cap (Fig. 3a–g), we hypothesized that the lamin A/C-mediated formation of an actin cap could resist the nuclear deformation resulting from external mechanical stimuli.

We found a lamin A/C-dependent distinct evolution of the nuclear morphology in response to substrate stretching (Fig. 3a–f). 3D-reconstructed confocal z-slices of the lamin B1-immunostained nuclei clearly showed that the lamin A/C-present WT MEFs formed the actin cap and their nuclei were flattened without changing the nuclear volume and surface texture (blue curves, Fig. 3g–k). To further confirm whether this nuclear volume conservation without surface wrinkling (i.e., nuclear protection) was lamin A/C-dependent, we compared the nuclear morphology of lamin A/C-bearing WT MEFs and lamin A/C-deficient *Lmna*^−/−^ MEFs (Fig. 3a–c vs. Fig. 3d–f). In contrast to the actin cap-forming WT MEFs that displayed gradual flattening of the nucleus (refer to *xz*-cross-sectional views, Fig. 3a–c), the surface texture of the nuclei of the actin cap-not-forming *Lmna*^−/−^ MEFs unexpectedly displayed severe deformation (Fig. 3d–f and red curves in Fig. 3g–k). This evolution of the nuclear morphology resulted in dramatic volume reduction as well as surface roughening (Fig. 3g–k). Accordingly, although the nuclear surface of the *Lmna*^−/−^ MEFs featured relatively weak deformation (Fig. 3d), as the substrate stretching progressed, surface folding was intensified (Fig. 3e), which finally evolved to deep wrinkles along the nuclear surface (Fig. 3f).

Together, these results revealed that the lamin A/C-mediated formation of an actin cap protects the nucleus from the extracellular mechanical stress and the loss of lamin A/C resulted in nuclear deformation.

### Molecular mechanism of actin cap-mediated nuclear protection

**Fig. 4:**
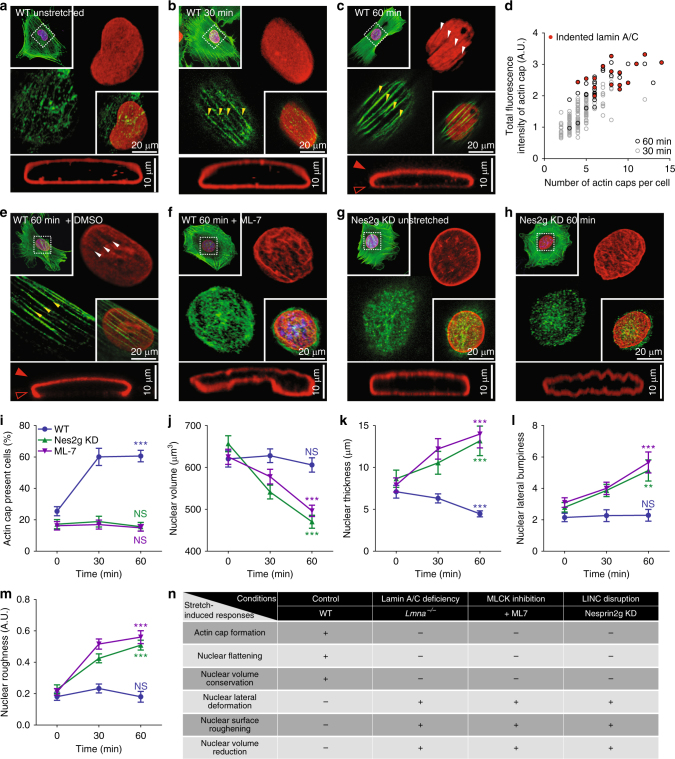
Underlying molecular mechanism of nuclear deformation. **a**–**d** Substrate stretching induced formation of an actin cap and reorganization of the nuclear lamin A/C. Although unstretched MEFs placed on the compliant PDMS film typically displayed a dismantled actin cap and vertically isotropic organization of the lamin A/C (red) wrapping the thick nucleus (**a**), continuous substrate stretching induces the formation of apical actin stress fibers (>30 min, **b**, **c**) and the nucleus became flattened (60 min, **c**). Note that the prolonged stretching time induces thickening of the stress fibers in the actin cap (**b**–**d**) and the formation of indented marks on the lamin A/C-stained apical surface of the nucleus along the actin cap (**c**, **d**), where lamin A/C forms an apically polarized dorm structure (**b** vs. **c**). Indented marks on lamin A/C were detected only after 60 min of stretching (**c**, **d**). **e**–**h** Two molecular mechanisms of stretch-induced nuclear morphological responses: actomyosin contractility and nucleus-cytoskeletal connectivity. Although lamin A/C was present, treatment of a myosin light-chain kinase (MLCK) inhibiting drug, ML-7 (**e** vs. **f**) and depletion of nesprin-2G (Nes2g) proteins (**g** vs. **h**) abrogated the stretching induced formation of an actin cap, where severe nuclear deformation was detected in response to substrate stretching (**f**, **h**). **i**–**m** Quantification of the actin cap formation and nuclear morphology of ML-7 treated (purple) and nesprin-2G depleted (green) MEFs in response to the substrate stretching. Note that the loss of actomyosin contractility or nucleus-cytoskeletal connectivity did not induce the formation of an actin cap (**i**) but reduced the nuclear volume (**j**), where severe nuclear morphological deformation was detected (**k**–**m**) in response to substrate stretching. **n** Summary of the relationship between the actin cap formation and nuclear morphological responses. In **i**, >150 cells were examined per condition and in **j**–**m**, >30 lamin B1-stained nuclei were analyzed per condition. Error bars represent S.E.M. of averaged values and statistical differences were calculated by unpaired *t*-test between unstretched control cells (0 min) and fully stretched cells (60 min). ***p < 0.0001, **p < 0.001, NS not significant (*p* > 0.05)

As we have shown that the substrate stretch-induced formation of an actin cap and nuclear volume conservation accompanied by nuclear flattening are regulated by lamin A/C (Fig. 3), we therefore hypothesized that the organization of lamin A/C could directly respond to the cytoskeletal tension applied to the nucleus. We previously showed that the 3D-organization of A-type lamins was interchangeable between an apically polarized dorm structure and an isotropic shell structure, depending on the actin cap-mediated cytoskeletal tension^18^. As predicted, the WT MEFs formed an isotropically distributed shell-structured lamin A/C before substrate stretching (i.e., no actin cap was formed in the unstretched PDMS films, Fig. 4a). After the cells were stretched, actin stress fibers appeared on top of the nucleus while lamin A/C still maintained a conventional dorm structure (Fig. 4b). However, the prolonged stretching induced thickening of the individual actin stress fibers, where dense accumulation of lamin A/C in the apical side of the nucleus was observed (Fig. 4c). Most strikingly, in this condition, we noticed indentation marks of lamin A/C along the individual actin cap fibers (Fig. 4c, d; Supplementary Figure 3). As the apical polarization of lamin A/C and indentation marks provide direct evidence that the actin cap transmits mechanical stress to the lamin A/C-bound nucleus^18,46,47^, our results further demonstrate that lamin A/C acts as a deformable molecular component that transmits stretch-induced extracellular mechanical stress to an intracellular biophysical force by precisely controlling the formation of the actin cap.

We now attempt to decipher the underlying molecular mechanism of the stretch-induced remodeling of the nuclear architecture. As mentioned above, nuclear flattening (Figs. 1g and 3i) and the formation of indented tracks on the apically polarized nuclear lamin A/C (Fig. 4c, d; Supplementary Figure 3) strongly suggested that the actin cap was not simply recruited by the substrate stretching but it could apply a pressing force vertically onto the nucleus. Moreover, the formation of a small number of thin fibers after 30 min of stretching did not significantly flatten the nucleus, but the prolonged stretching induced the apically polarized nuclear lamin organization (Fig. 4c, d, k). We therefore tested if actomyosin contractility would be required to mediate the stretch-induced remodeling of nuclear lamin A/C. We treated lamin A/C-present WT cells with ML-7, a myosin light-chain kinase (MLCK) inhibiting drug. Compared to ML-7-untreated DMSO control cells, MLCK-inhibited (i.e., myosin-based contractility lost-) cells did not form an actin cap, and thus apically polarized lamin A/C was not detected in response to substrate stretching (Fig. 4e–f). Accordingly, their nuclear morphology was not protected, i.e., the nuclear volume was reduced and the nuclear surface was deformed (Fig. 4j–m).

As lamin A/C-anchored LINC complexes mediate a physical interplay between the nuclear envelope and the actin cytoskeleton^48^, we then tested nesprin-2G depleted cells as a cell model deficient of LINC-mediated connectivity between the cytoskeleton and the nucleus. Unstretched nesprin-2G depleted MEFs showed a largely similar morphology to the unstretched WT cells, featuring the absence of the actin cap and isotropic shell-structured lamin A/C (Fig. 4a vs. g). Under stretched condition, however, compared to the actin cap-forming WT cells that maintained the nuclear volume and resisted nuclear deformation by nuclear flattening, the nesprin-2G-depleted cells did not form the actin cap, and thus their lamin A/C was not vertically reorganized, and the nucleus was highly deformed and displayed a folded surface texture (Fig. 4g, j, i–m).

Therefore, the alteration of the nuclear morphology was not a trivial result, but a consequence of the lamin A/C-mediated formation of an actin cap, where the LINC-mediated nucleus-cytoskeletal connection and actomyosin-based contractility of the actin cap underlie the force-responding molecular mechanism of lamin A/C (Fig. 4n). Together with our previous results that demonstrated that nuclear volume reduction and surface bifurcation were the consequence of the disruption of the cytoskeleton-mediated force balance between the nucleus and cytoplasm^23^, these results show the following biophysical responses: (i) substrate stretch-induced mechanotransduction activates the formation of an actin cap only in the cells that contain lamin A/C, (ii) extracellular mechanical stimuli disrupt nuclear morphology in lamin A/C-deficient cells, (iii) actin cap-forming lamin A/C-present cells maintain nuclear volume by applying compressional forces on the nucleus, and (iv) this nuclear protection by the actin cap relies on LINC-mediated nucleus-cytoskeletal connections and actomyosin contractility.

### Actin cap reduces nuclear stress under stretching

**Fig. 5:**
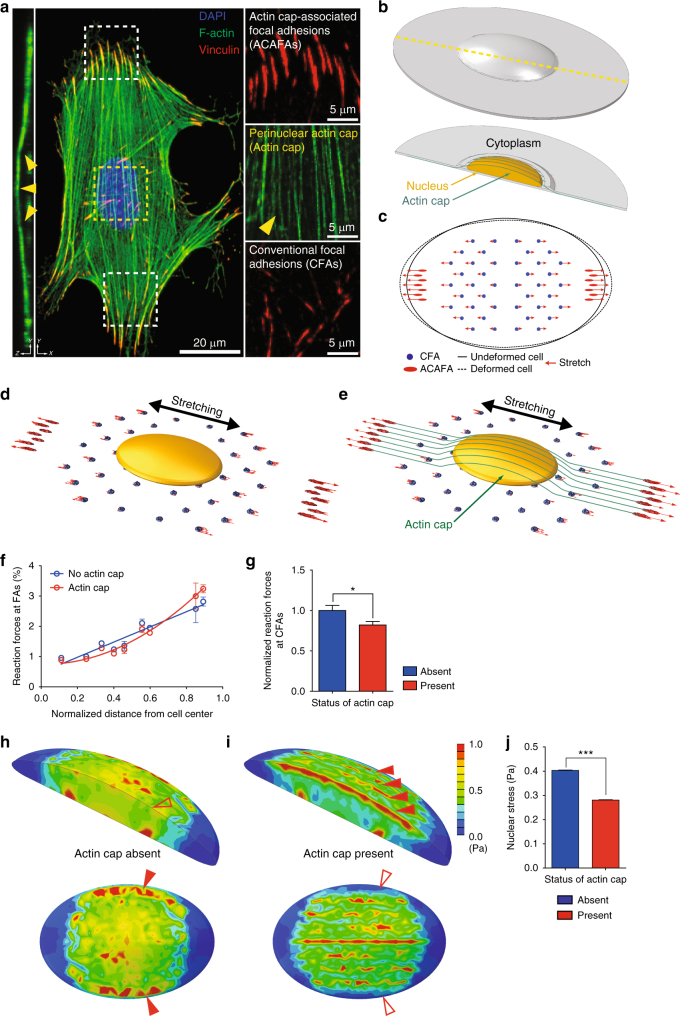
Actin cap induced reduction of nuclear stress by focusing focal-adhesion forces on the periphery of the cell. **a** Organization of an actin cap and actin cap-associated focal adhesions in a MEF. Nucleus (blue), F-actin (green), and vinculin-stained focal adhesions (red) were visualized by maximum intensity projection of the *z*-stacked immunofluorescence confocal images. Vertical cross-section depicts the characteristic topology of the actin cap. Parallel-aligned actin cap (yellow arrowhead) are terminated by actin cap-associated focal adhesions (ACAFAs) at the basal surface of the cell, which are larger than the conventional focal adhesions (CFAs). **b**, **c** 3D finite element model of an adherent MEF. Cross-section (yellow dotted line) of continuum solid elements for cytoplasm reveals a perinuclear region, a continuum solid for nucleus, and 3D beams of the actin cap (**b**). Red arrows in projected bottom view correspond to the linear stretch equivalent to 8% strain (**c**). **d**–**g** Effects of the actin cap on the forces acting on focal adhesions under stretching. Vector results of the force in the focal adhesions are depicted in the cell model in the absence (**d**) and presence (**e**) of an actin cap. Radial distribution of reaction forces at focal adhesions (**f**) and the average values (**g**) were calculated depending on the formation of an actin cap in **f** and **g**, 56 focal adhesions included in the cell model were assessed. **h**–**j** Nuclear stress distribution in the stretched cell. Isometrics of von-Mises stresses in the absence (**h**) and presence (**i**) of an actin cap reveal that external forces are ~30% less transferred to the nucleus of the actin cap forming cell than the actin cap absent cell (**j**). In **h** and **i**, the cross-sectioned nuclei were color-coded to clearly show the stress distribution, where scale bars indicate the magnitude of mises stresses. In **j**, to facilitate quantitative comparison, nuclear stress was expressed as von-Mises stress that represent a non-directional scalar quantity. Values were extracted from 19,248 different nodes inside the nucleus. In **f**, **g**, and **j** statistical analyses were performed using the unpaired *t*-test, where error bars represent S.E.M. of averaged values; ***p < 0.0001, *p < 0.05

To scrutinize the underlying mechanics governing the cellular mechanical homeostasis (i.e., intracellular force balance between nucleus and stretch-induced cytoskeletal tension), we introduced a computer-aided cell modeling method. We first developed a finite element model for the whole cytoplasm based on previous studies on physical properties^49–53^ and dimensional analysis of the cell components^27,51,54,55^ (Supplementary Table 1). This continuum solid elements model developed in our study includes a nucleus, actin stress fibers, and focal adhesions (Fig. 5a–c). Owing to their distinct topology, actin stress fibers in the perinuclear region mimicking actin cap fibers are under higher tension than conventional basal stress fibers^17^. Hence their terminating focal adhesions in the basal surface of the cell, i.e., actin cap-associated focal adhesions (denoted by ACAFAs, Fig. 5a) are larger and more sensitive to changes in substrate compliance than regular focal adhesions, i.e., conventional focal adhesions (denoted by CFAs, Fig. 5a)^14,16^.

We now simulate the stress distribution within the cell by applying tensile stress to the cell model in the presence and absence of the actin cap and examining the reaction forces developed at focal adhesions (Fig. 5b–g). The finite element model incorporated with nucleus, actin cap fibers, and cytoplasm was deformed in 8% strain ratio as applied in actual experiments, which resulted in elongation of cell in the direction of stretching (Fig. 5b, c). Simulation of this model with and without actin cap enabled the analysis of actin cap-dependent force distribution in the cell (Fig. 5d, e). We found that the reaction forces became concentrated at actin cap-associated focal adhesions, specifically located near the cell periphery, where the actin cap-associated focal adhesions accounted for a greater percentage of the total reaction force than conventional focal adhesions (Fig. 5f). Consequently, the percentage of reaction forces acting on conventional focal adhesions that occupied the majority of the focal adhesions in a cell was reduced by >20% in the presence of the actin cap compared to the actin cap absent counterpart (Fig. 5g). These results demonstrate that the actin cap reduces the mechanical stresses loaded on the cytoplasm by reallocating the reaction forces at the actin cap-anchoring focal adhesions.

So far, we have discovered that the formation of an actin cap was induced by substrate stretching in lamin A/C-present control cells but not in lamin A/C absent cells (Figs. 1–3), and this mechano-response prevented nuclear deformation (Figs. 3 and 4) by accumulating the majority of reaction forces in the actin cap-associated focal adhesions (Fig. 5f, g). To generalize our structure-based functional interplay model that supports the cell-stretching experimental results, we finally asked if the alteration of the nuclear morphology was the consequence of the mechanical disturbance applied from the extracellular stimuli.

To address this query, we compared the distribution of subcellular forces acting on the nucleus in response to tensile stress applying to the lamin A/C-bearing control cells, with and without an actin cap (Fig. 5h–j). As external stresses acting on the cytoplasm were directly transferred to the nucleus, in the actin cap absent cell, stresses acting on the nucleus were largely elevated all around the nucleus (Fig. 5h), which could promote significant nuclear deformation (Supplementary Table 2). However, in the actin cap present cell, the stress acting on the whole nucleus was significantly reduced (>30%, Fig. 5h–j). Accordingly, the driving force to deform the nucleus was reduced, and thus nucleus was less deformed than the cell model missing the actin cap (Supplementary Table 2).

In addition, we noted concentrated stresses along the nuclear boundary in the actin cap absent cell (red arrowheads, Fig. 5h), which was induced by direct contact with the cytoplasm deformed by external stresses. This result further confirms the nuclear deformation observed in the actin cap-not-forming lamin A/C absent cells that featured lateral compression of the nucleus (Fig. 3d–f). On the other hand, narrow regions representing the enhanced stress appeared on the apical side of the nucleus along the actin cap fibers in the actin cap present cell (red arrowheads, Fig. 5i). These localized regions were attributed to the confinement of the compliant nucleoplasm due to the load-carrying apical actin cables (i.e., actin cap fibers) and their terminating focal adhesions (i.e., actin cap-associated focal adhesions). The relocation of the stresses by actin cap fibers control the direction of the force applied to the nucleus, which explains the nuclear flattening in the actin cap-forming WT cells (Fig. 3a–c) and indentation marks on the apical surface of the nucleus (Fig. 4a–c; Supplementary Figure 3). Consequently, this simulation demonstrates the critical role of the actin cap in maintenance of the nuclear morphology in response to the external mechanical stimuli: (i) extracellular mechanical stress transferred to the nucleus via cytoplasm deforms the nuclear morphology, (ii) but the elevated nuclear stress could be alleviated by the formation of an actin cap that concentrates those forces only the small regions along the actin cap.

**Fig. 6:**
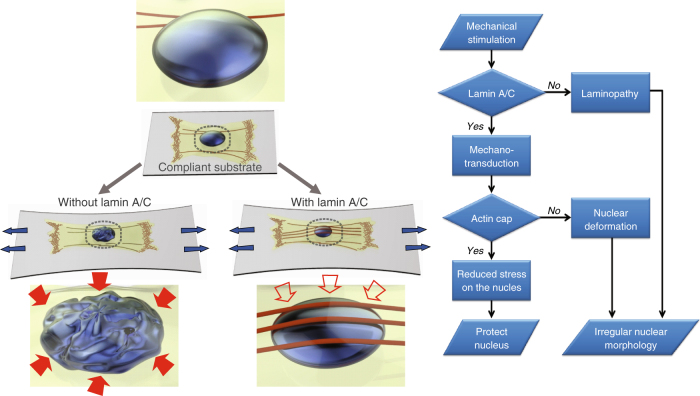
Summary of the functional interplay between lamin A/C and actin cap in response to the external physical stimuli. A schematic flow chart of the lamin A/C-mediated cellular mechanotransduction and the role of the actin cap in protecting nuclear morphology. The external mechanical stimuli trigger the formation of actin cap in lamin A/C-present cells, which reduces the stress transferred to the nucleus and maintains the nuclear morphology. Laminopathic cells featuring the loss of the actin cap typically display the irregular nuclear morphology

Together, our computational analyses confirm that the lamin A/C-mediated formation of an actin cap protects the nucleus by dissipating stresses directly acting on the nucleus, which provides physiological evidence of abrogated mechanotransduction in laminopathic diseases models (summarized in Fig. 6).

## Discussion

The combination of biological experiments and computational simulation reveals that the critical role of the actin cap in protecting the nucleus from the extracellular mechanical stimuli is mediated by the nuclear lamin A/C. We previously showed that shear flow induces the formation of the perinuclear actin cap, much more rapidly than biochemical stimulation with serum, termed actin cap-mediated mechanotransduction^56^. Cellular mechanosensation of substrate compliance is also mediated by actin cap-associated focal adhesions that terminate both ends of the actin stress fibers at the basal side of the cell^16^. Our new results unveil the underlying mechanism of the finely orchestrated cellular responses to the extracellular stimuli, where the lamin A/C-mediated formation of an actin cap prevents nuclear deformation in response to the substrate stretching. In cells featuring lamin A/C, the stretch-induced formation of a perinuclear actin cap had a significant role in reducing nuclear stress by pressing down the nucleus.

To identify the underlying mechanics governing the cellular mechanical homeostasis (i.e., intracellular force balance), we constructed a cell model, which enabled us to investigate the way in which the lamin A/C-mediated actin cap could protect the nucleus, i.e., why the actin cap is formed to protect nuclear morphology from mechanical stress (Fig. 5). By simulating stress distribution within the cell in response to the mechanical stimulation, we identified the load-carrying mechanism of the actin cap. Computational modeling and simulation revealed that the perinuclear apical actin cables corresponding to the actin cap could carry most of the tension applied to the cell, which helped the nucleus to resist external mechanical disturbances and thus to maintain its intact morphology. Close observations of the cytoskeleton-dependent nuclear reshaping such as nuclear flattening^18^ and nuclear surface indentation^46^ also support our discovery of the nuclear stress-cancellation by the perinuclear actin cap, which so far has not been identified.

Lamin A/C mutations, however, lead to defective gene transcription and imperfect nuclear mechanics^57^, which could possibly attenuate the molecular anchorage of the actin cap to the nuclear lamin^58–60^. Therefore, in cells lacking lamin A/C, due to the absence of the actin cap, an external mechanical signal is transferred to the whole cytoplasm and the nucleus consequently becomes subject to the enhanced stress level (Fig. 5j). Together with the previous experimental approaches that revealed the mechano-transducing role of the actin cap^13,14,56^, our work further confirms that the actin cap acts as the critical load-carrying component of the mechanically stressed cell that requires lamin A/C to compose the mechanical pair with the actin cap.

In our model, the actin stress fibers composing the actin cap were modeled as discrete shear deformable beams with three-node line elements based on the Timoshenko beam theory^61^, where bending members could experience the significant flexural and shear deformations that were largely neglected by the classical beam theories. The refined formulation provided by Timoshenko beam theory offered a high-order beam element that accounts for both flexural and shear deformation effects on the single stress fiber in this study. Therefore, our cell model provided an accurate prediction of the actin cap-mediated responses to the mechanical stimuli.

The presence of lamin A/C radically changed the manner in which the cell accommodated the external mechanical stimulation. Cells whose actin cap was physically connected to the nucleus (i.e., WT MEFs) not only decreased the deformation of the nucleus by reducing the stresses applied to the nucleus, but also increased the longitudinal stiffness of the cell due to the marked concentration of forces on the focal adhesions terminating both ends of actin cap fibers (Fig. 5d–f). Moreover, the majority of the cells that lost the physical connection between the nucleus and actin cap (i.e., *Lmna*^−/−^ MEFs) did not form an actin cap (<10%, Fig. 3g), where neither redistribution of nuclear stress nor protection of nuclear morphology was available (Figs. 3 and 5). Together with these simulation results, our discovery—the reduction of nuclear stress by the actin cap—could therefore provide a novel mechanopathological perspective on the structural vulnerability of laminopathic cells that typically do not form an actin cap, have lower nuclear stiffness than normal cells, and thus display an irregular nuclear shape.

This work elucidated the importance of the unique topology of an actin cap that physically connects the apical side of the nucleus (via LINC complexes) to the basal side of the cell (via actin cap-associated focal adhesions), where the actin cap serves as a conduction path of the mechanical signal and simultaneously discharges the stresses transferring onto the nucleus. Our experimental data clearly supported this notion: (i) *Lmna*^−/−^ MEFs did not form an actin cap while cells were exposed to continuous stretching (Fig. 3) and (ii) a fully developed actin cap that could apply forces on the nuclear surface was observed after sufficient stretching of *Lmna*^+/+^ MEFs (Fig. 4c, d). In addition to the experimental data delineating the role of lamin A/C in the formation of the mechanical load-carrying actin cap, this notion was also supported by the following results by the computational modeling and simulation: (i) altered distribution of focal-adhesion forces (i.e., more concentrated in actin cap-associated focal adhesions) and (ii) decreased deformations and stresses on the nucleus of the actin cap resent cell model.

The irregular nuclear morphology in various severe genetic disorders, collectively termed laminopathies, is attributed to the abnormalities in the nuclear lamina mainly caused by the mutation of *LMNA*^41^. On the basis of these findings, it has been suggested that the changes in nuclear shape are intimately linked to the cell functions in physiological and pathological processes as well as during development. Therefore, this work explains why the substrate stretching induced mechanical stress triggers the formation of the actin cap, where the formation of an actin cap in lamin A/C-bearing cells (i.e., normal healthy cells) is a more favorable condition than in cells lacking an actin cap to prevent nuclear structural abnormality.

## Methods

### Cell culture and immunofluorescence

MEFs were cultured in Dulbecco’s Modified Eagle’s Medium (DMEM, ATCC) supplemented with 10% fetal bovine serum (FBS, ATCC), 100 U/ml penicillin, and 100 μg/ml streptomycin (Sigma) at 37 °C with 5% CO_2_ in a humidified incubator, as reported previously^13,16,18,20,23,62^. After allowing the cells to grow on a collagen pre-coated glass bottom dish (MatTek) for 6 h, they were fixed with 2% paraformaldehyde (Sigma-Aldrich) for 1 h and permeabilized with 0.1% Triton X-100 (Fisher biotech) for 10 min, and then blocked with phosphate-buffered saline (PBS) supplemented with FBS (10%, v/v) for 20 min. The nucleus, nuclear lamina, actin stress fibers, and focal adhesions were immunostained with DAPI (300 nm, Invitrogen), anti-lamin A/C antibody (1:200, EMD Millipore, MAB3538), anti-lamin B1 antibody (1:500, abcam, ab8982), Alexa-Fluor phalloidin (1:40, Invitrogen), and anti-vinculin antibody (1:500, Sigma-Aldrich, V4505), respectively. All images were captured using confocal laser microscopy (A1R, Nikon) through a ×20 plan lens (N.A. 0.5) or ×60 plan lens (N.A. 1.2).

### Transient transfection and live cell imaging

GFP–lamin-A was transfected to monitor the nuclear morphology and volume, and EGFP–LifeAct was transfected to visualize actin cytoskeleton that also allows the measurement of cell orientation. The transient transfection complex was prepared in Opti-MEM I reduced serum medium (Gibco, Carlsbad, CA), where FuGENE® HD (Roche, Indianapolis, IN) was mixed as a transfection agent following the manufacturer’s instructions. Confocal microscopy of live cells was conducted using a ×60 Plan Fluor lens (Nikon, N.A. 1.4). Each frame was taken every 4 to 20 min to avoid significant photobleaching during the imaging time of up to 2 h. *Z*-stacked time-lapsed confocal images were three-dimensionally reconstructed and the 3D rendered images were analyzed using NIS elements software (Nikon).

### Substrate deformation

Sylgard 184 silicon elastomer base and curing agent (Dow Corning) were combined at 10:1 ratio by mass and mixed vigorously. After desiccation until all bubbles disappeared, the solution was poured into a 10 cm petri dish. To obtain a uniformly spread thin layer of the solution, the petri dish was spin-coated for 20 s at 500 rpm before baking in an oven for 1 h at 50–60 °C. Cured elastic membranes were carefully carved out to fit into the custom-made stretching device, sonicated in ethanol, and then washed with DI water. The substrate stretching device was designed to stretch cells placed onto the elastic membranes in a uniaxial direction, as a modification of the previous design^63^. Briefly, the substrate stretching device consisted of a metal dish with a glass coverslip-sealed hole in the bottom, which enabled monitoring of the cells seeded on the transparent elastic thin film while it was stretched. Silicone oil was added between the thin film and the glass coverslip as a lubricant and film stretching was controlled by negative pressure exerted by a vacuum pump (Supplementary Figure 1A). The film was stretched 8% in the uniaxial direction and stretching and relaxation were subject to repeated vacuum loading at 1 Hz. This value was consistently applied to all the experiments in this work after careful monitoring cell responses in various stretching frequency and duration (Supplementary Figure 1B, C). After assembling all the parts, the elastic thin film inside the device was coated with 0.2 mg/ml rat tail type-I collagen (BD Biosciences) diluted in 0.2 N acetic acid before plating the cells. After gently washing the film with PBS, the cells were plated onto the cell culture media added substrate stretching device and incubated for at least 6 h to secure cell attachment before stretching the elastic film.

### Quantification of nuclear texture and volume

We estimated the surface roughness of the 3D-reconstructed nuclei using a customized program coded in MATLAB (MathWorks Laboratory, Natick, MA). To represent the overall shape of the nucleus, we performed a morphometric image analysis in three different *z*-positions corresponding to 25, 50, and 75% of the total height of the nucleus, each denoted by the top, middle, and bottom of the nucleus, respectively. In each selected height, the distance between the center and the boundary of each nucleus was plotted as a function of degrees in a clockwise direction, which was termed the ‘amplitude profile’. To eliminate noise stemming from the analysis of pixelated images, the Savitzky–Golay filtering of 6 degrees was applied to mathematically smooth the data, where we quantified local maximum values represented by peaks in the plot. The height variation of amplitude profile obtained from the vertical cross-sectional image of 3D-reconstructed nucleus was normalized by the lateral length of the scanned nuclear surface to quantify the nuclear surface roughness.

Nuclear volume measurement was performed using a customized MATLAB code as previously described^23^. Briefly, *z*-stacks of confocal images of GFP–lamin A was 3D-reconstructed to localize the outer surface of nuclear envelope, where the outer surface was defined to be independent of the threshold value. 3D domain of pixels within the outer surface of the nucleus was integrated to obtain the total nuclear volume. This approach was validated by measuring the volumes of spherical fluorescence microspheres (2 µm in diameter, Invitrogen) using the same confocal microscopy (A1R, Nikon).

### Computational model of cells under stretch

A 3D finite element model (FEM) of adherent single cells was developed to study the mechanical interactions among subcellular components and, more specifically, the role of actin cap fibers in the mechanical behavior of cells. The cell model included three phases—the cytoplasm, the nucleus, and actin cap fibers, whose mechanical properties were experimentally measured previously (Supplementary Table 1). Implicit in the use of the mechanical properties is the assumption of time-independent elasticity. Although this assumption is useful for reasonably short time scales and for relative comparisons within the model response, it ignores the longer term viscoelastic and viscoplastic nature of the constituent parts. In addition, our model was designed to understand mechanical behavior of the cell at the micro/nanometer level, so it was computed in static analysis to obtain intuitive results by eliminating physical factors such as rotary inertia that makes the interpretation complicated. Modeling and simulation were performed in the general-purpose finite element analysis (FEA) software ABAQUS version 6.10 (Dassault Systemes).

The cytoplasmic perinuclear regions and the nucleus were modeled as solid deformable continua with ten-node tetrahedral elements (C3D10M), which are 3D stress-displacement elements appropriate for meshing complex shapes with excellent contact properties. The actin cap fibers were modeled as discrete shear deformable beams with three-node line elements (B32) based on the Timoshenko beam theory^61^. The number of actin caps used in this model was averaged from our previous publications^12–14,16,18,56^ and thus, seven actin cap fibers were included and modeled to restrict their movement within the cytoplasm. The dimensions of each component included in the finite element model of the cell were determined based on a simplified idealization of the typical morphology of human and rodent fibroblasts (Supplementary Table 1). Methods for connecting the material properties of the model to experiments have been pursued in an ad hoc fashion^26^ and more recently by using established material homogenization techniques^29^. In this model, the surfaces of nucleus, cytoplasm and actin cap fibers are in contact with each other. All the contact points were assumed frictionless in the tangential direction to avoid the effect of unknown parameters. In the model, external mechanical stimulation acting on the cell was imposed through the focal adhesions, akin to stretching the compliant membrane in an experiment. The dimension, morphology, and location of focal adhesions have been determined previously^16^, where circular-shaped conventional focal adhesions (2.3 μm^2^, 42 per cell) were uniformly distributed on the basal plane of the cell and actin cap-associated focal adhesions terminating the actin cap fibers had an elliptical shape with major and minor diameters of 4 and 1μm, respectively. The actin cap fibers were exclusively localized in two narrow sectors of the cell periphery. Tension was introduced to our finite element model via simulation of a stretch experiment, whereby a linear displacement equivalent to 8% strain was applied at the focal adhesions. The ends of actin cap elements were connected directly to the corresponding actin cap associated focal adhesions using tie constraints in ABAQUS. In the basal plane of the cell, longitudinal in-plane movements were prescribed at the focal adhesions, whereas transverse in-plane movements were free at the focal adhesions. All other points in the basal plane were free to move in-plane, but out-of-plane movement was restricted.

### Data availability

All the data are available from the corresponding author on reasonable request.

## Electronic supplementary material

Supplementary Information

Peer Review File

Description of Additional Supplementary Files

Supplementary Movie 1
