# Author Correction: Nuclear lamin A/C harnesses the perinuclear apical actin cables to protect nuclear morphology

**DOI:** 10.1038/s41467-018-03450-2

**Published:** 2018-03-13

**Authors:** Jeong-Ki Kim, Arghavan Louhghalam, Geonhui Lee, Benjamin W. Schafer, Denis Wirtz, Dong-Hwee Kim

**Affiliations:** 1 KU-KIST Graduate School of Converging Science and Technology, Korea University, Seoul, 02841 South Korea; 2 Department of Civil and Environmental Engineering, University of Massachusetts Dartmouth, Dartmouth, MA 02747 USA; 3 Institute for NanoBioTechnology, The John Hopkins University, Baltimore, MD 21218 USA; 4 Department of Civil Engineering, The John Hopkins University, Baltimore, MD 21218 USA; 5 Department of Chemical and Biomolecular Engineering, The John Hopkins University, Baltimore, MD 21218 USA; 6 Johns Hopkins Physical Sciences—Oncology Center, The Johns Hopkins University, Baltimore, MD 21218 USA; 7 Departments of Pathology and Oncology and Sydney Kimmel Comprehensive Cancer Center, The Johns Hopkins School of Medicine, Baltimore, MD 21205 USA

Correction to: *Nature Communications* 10.1038/s41467-017-02217-5, published online 14 December 2017

In the original version of this Article, the affiliation details for Arghavan Louhghalam were incorrectly given as ‘Institute for NanoBioTechnology, The Johns Hopkins University, Baltimore, MD, 21218, USA’, and it should have been given as ‘Department of Civil and Environmental Engineering, University of Massachusetts Dartmouth, Dartmouth, MA 02747, USA’. Furthermore, an incorrect grant number, R1610512, was acknowledged. The correct grant number is NRF-2016R1C1B2015018. These errors have now been corrected in both the PDF and HTML versions of the Article.

