# Supplementary material for: Nuclear lamin A/C harnesses the perinuclear apical actin cables to protect nuclear morphology
Source: Nat Commun. 2017 Dec 14;8:2123. doi: 10.1038/s41467-017-02217-5 (PMC5730574; doi:10.1038/s41467-017-02217-5)
Supplement: Supplementary file 1 — Supplementary Information [file 41467_2017_2217_MOESM1_ESM.pdf]

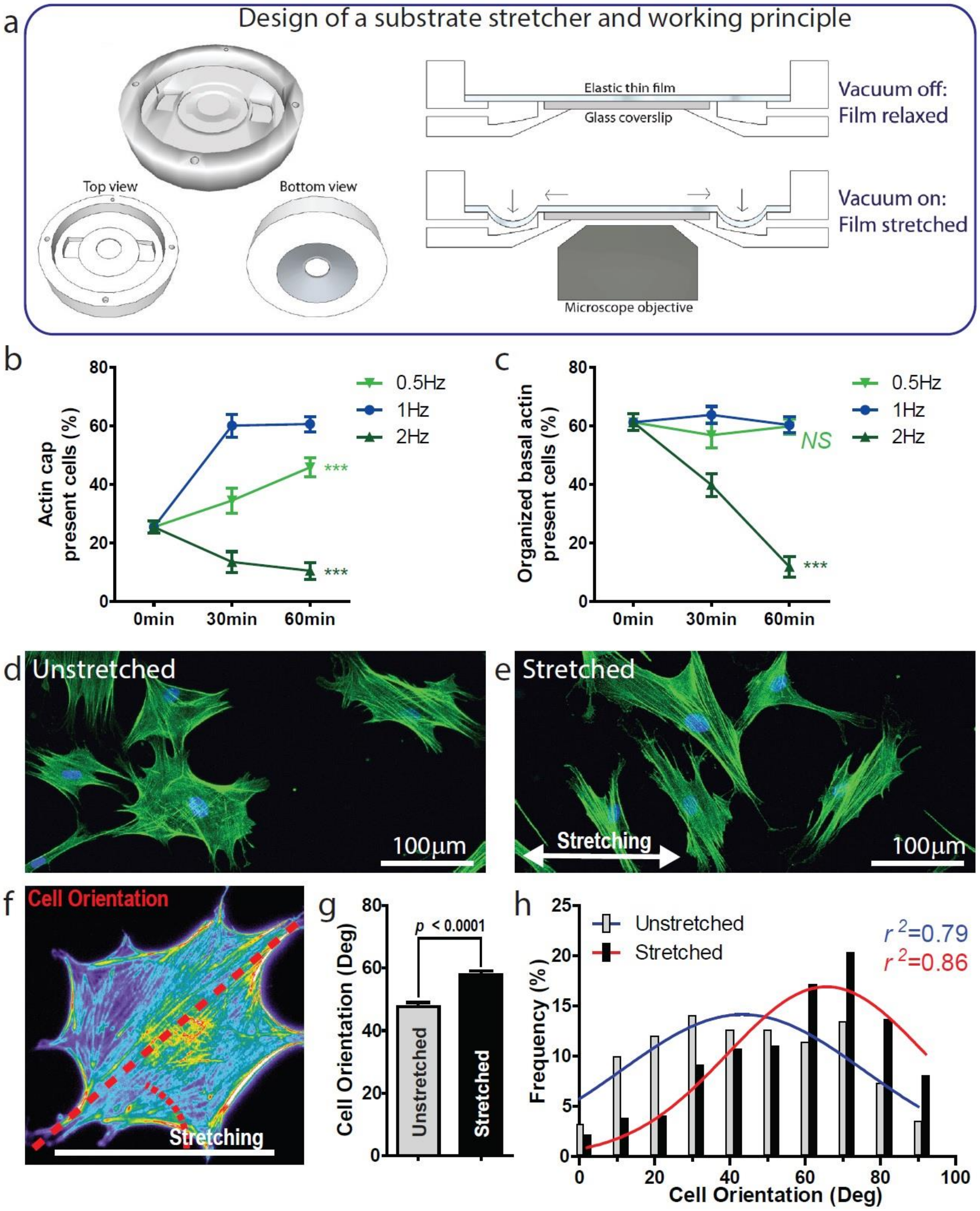

Supplementary Figure 1. Verification of stretch-induced cellular responses. a. Schematic of the substrate stretcher. **b-c.** Stretch-induced actin organization. In panels b and c, >150 cells were examined per condition to assess the formation of an actin cap (b) and basal actin stress fibers (c). Error bars represent the S.E.M. of averaged values. Statistical differences were calculated by unpaired *t*-test between unstretched control cells (0min) and fully stretched cells (60min). ***: $p<0.0001$, *NS*: not significant ($p>0.05$). Refer to **Figure 3**, **Supplementary Figure 2**, and **Supplementary Note 1** for the detailed information for the formation of actin cap and basal actin structure. **d-h.** Cell re-orientation under uniaxial cyclic stretching of the substrate. Randomly seeded mouse embryonic fibroblasts (MEFs) on the stretchable PDMS thin film were re-oriented in response to the substrate stretching by the realignment of cell body perpendicular to the substrate stretching direction (d, e, f). The distribution of cell-orientation angles better followed Gaussian statistics after substrate stretching (g, h). Statistical comparison of cell orientation angle between unstretched control cells and stretched cells represents the significant reorientation of the cells perpendicular to the stretching direction, where error bars indicate S.E.M., statistical differences were calculated using the unpaired *t* test ($p<0.0001$), and coefficients of determination (r^2) of color-coded Gaussian fits are shown in each plot (g-h).

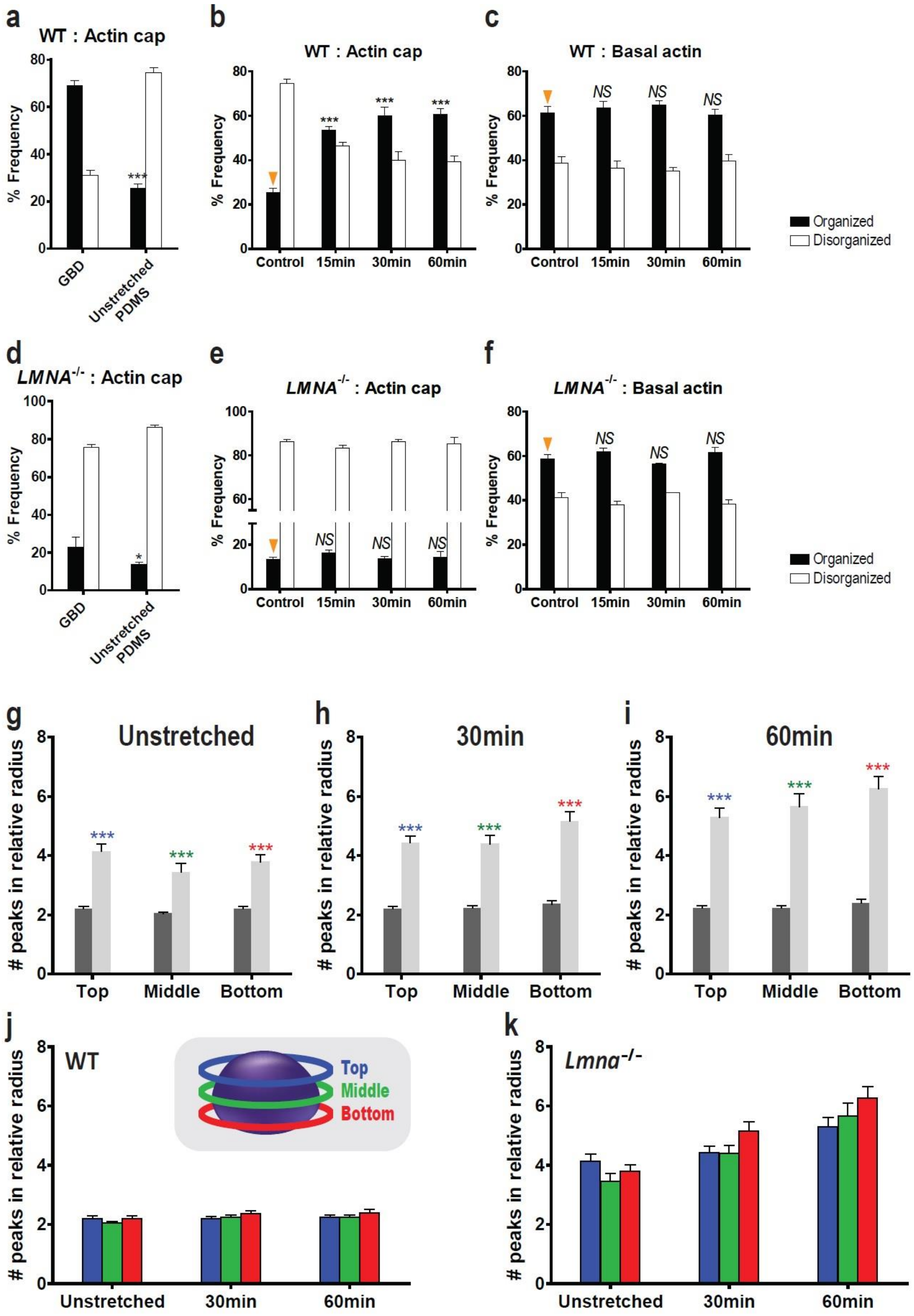

Supplementary Figure 2. F-actin organization and nuclear morphology in WT and *Lmna*$^{-/-}$ MEFs. a-f. Effects of PDMS substrates, lamin A/C, and stretching duration on the formation of an actin cap. In panels a-f, proportions of cells corresponding to organized (black) and disorganized (white) organization of the actin cap (a, b, d, e) or basal actin stress fibers (c, f) were quantified in WT (a-c) and *Lmna*$^{-/-}$ (d-f) MEFs. In panel a-f, >150 cells were examined per condition and error bars represent S.E.M. of averaged values. In panels b, c, e, and f, 1-way ANOVA using Dunnett's *post test* was applied based on the values in the unstretched control (marked by an inverted triangle) for the multiple comparisons of the fraction of cells forming an organized actin cap or an organized basal actin structure; ***: $p<0.0001$, *NS*: not significant ($p>0.05$). **g-k.** Comparision of nuclear morphology between WT and *Lmna*$^{-/-}$ MEFs in response to substrate stretching. Nuclear lateral bumpiness of *Lmna*$^{-/-}$ MEFs is larger than WT before and after substrate stretching. In panels g-k, >30 cells were analyzed per condition; ***: $p<0.0001$.

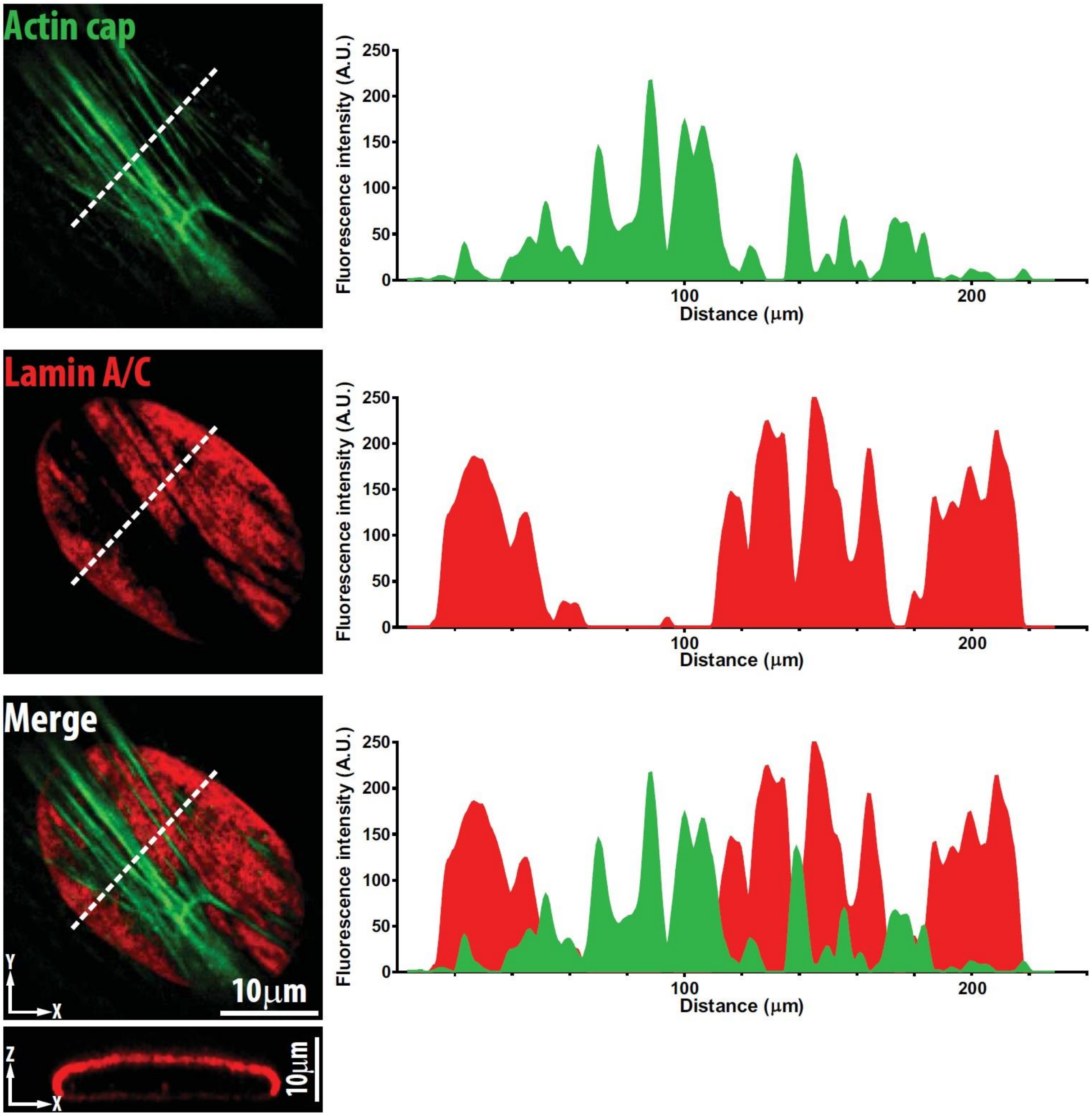

Supplementary Figure 3. Details of indentation marks formed on the nuclear lamin A/C. An apically polarized dorm structured lamin A/C was organized after 60 min of substrate stretching and the enhanced cytoskeletal tension due to the actin cap forms indented marks along the actin cap in the apical side of the lamin A/C stained nucleus. Scanning of fluorescence intensity of F-actin (green) and lamin A/C (red) along the fixed line (white broken lines) demonstrates the actin-cap-induced lamin A/C indentation.

Supplementary Table 1.

Mechanical properties and dimensions used in finite element model

Description	Value	Reference
Shear modulus of the cytoplasm (G_c)	85 Pa	[1]
Shear modulus of the nucleus (G_n)	50 Pa	[2]
Shear modulus of the nuclear lamina (G_l)	50 Pa	[2]
Elastic modulus of actin-cap fibers (E_f)	10 kPa	[3]
Poisson's ratio of the cytoplasm (v_c) [#]	0.4	[2,4,5]
Poisson's ratio of the nucleus (v_n) [#]	0.4	[2,4,5]
Poisson's ratio of the nuclear lamina (v_l) [#]	0.4	[2,4,5]
Approximate dimension of the nucleus	30×20×5μm	[6]
Approximate dimension of the cytoplasm	90×60×7μm	[7]
Radius of actin cap stress fibers	100 nm	[3]

[#] *: Fixed Poisson ratio of cellular components was used for simplicity*

Supplementary Table 2.

Simulated nuclear deformation

Nuclear dimensional changes	Stretch-induced nuclear deformation		
	Actin cap absent	Actin cap present	*Δ**
Lateral<br>*(X' – X)*	1.328μm	0.856μm	- 36%
Vertical<br>*(Y' – Y)*	0.110μm	0.031μm	- 72%

**: Δ = % change of nuclear deformation in the presence of actin cap*

Supplementary Note 1

(1) **A microscope-mounted substrate stretcher and its working principle.** A circular hole in the center of the device was sealed with a cover glass through which cells were visualized on top of the microscope object lens. Stretching of cells seeded on the PDMS film was controlled by switching the vacuum pump. To find the optimum frequency of substrate stretching in a fixed strain ratio (i.e., 8%), actin organization was monitored in different stretching frequencies (0Hz, 1Hz, and 2Hz). While increasing the stretching frequency tended to promote the formation of an organized apical actin stress fibers (from 0.5Hz to 1Hz), this change was not maintained until 2Hz of stretching. This behavior was attributed to the cell detachment or cell death in such a high frequency and accordingly, the population of cells that formed organized basal actin structure was significantly reduced in the 2Hz of highly stretched condition. Therefore, all the experiments in this work were performed in the 8%, 1Hz of substrate stretching condition.

(2) Additional observation of cell re-orientation under uniaxial cyclic stretching of the substrate. Randomly seeded mouse embryonic fibroblasts (MEFs) on the stretchable PDMS thin film were re-oriented in response to the substrate stretching by the realignment of cell body perpendicular to the substrate stretching direction (Supplementary Figure 1D, E). Cell orientation angle (θ) was defined as the acute-angle between the longest chord through the cell (marked by a dashed line) and the substrate stretching direction (marked by an arrow), approaching 0° for parallel-aligned cells and 90° for cells perpendicularly aligned to the substrate stretching direction. The distribution of cell-orientation angles better followed Gaussian statistics after substrate stretching (see the color-coded regression curves and corresponding r^2 values, Supplementary Figure 1 G, H), which further indicated that stretched cells were realigned more uniformly perpendicular to the substrate stretching direction.

Supplementary References

1 Yamada, S., Wirtz, D. & Kuo, S. C. Mechanics of living cells measured by laser tracking microrheology. *Biophys J* **78**, 1736-1747, doi:doi:10.1016/S0006-3495(00)76725-7 (2000).
2 Panorchan, P., Schafer, B. W., Wirtz, D. & Tseng, Y. Nuclear envelope breakdown requires overcoming the mechanical integrity of the nuclear lamina. *J Biol Chem* **279**, 43462-43467, doi:DOI 10.1074/jbc.M402474200 (2004).
3 Lu, L., Oswald, S. J., Ngu, H. & Yin, F. C.-P. Mechanical properties of actin stress fibers in living cells. *Biophys J* **95**, 6060-6071 (2008).
4 Ethier, C. R. & Simmons, C. A. Introductory Biomechanics: From Cells to Organisms. *Camb Text Biomed Eng*, 1-511, doi:Doi 10.2277/ 0521841127 (2007).
5 Vaziri, A., Lee, H. & Mofrad, M. R. K. Deformation of the cell nucleus under indentation: Mechanics and mechanisms. *J Mater Res* **21**, 2126-2135, doi:Doi 10.1557/Jmr.2006.0262 (2006).
6 Tseng, Y., Schafer, B. W., Almo, S. C. & Wirtz, D. Functional synergy of actin filament cross-linking proteins. *J Biol Chem* **277**, 25609-25616, doi:DOI 10.1074/jbc.M202609200 (2002).
7 Heidemann, S. R. & Wirtz, D. Towards a regional approach to cell mechanics. *Trends in cell biology* **14**, 160-166, doi:10.1016/j.tcb.2004.02.003 (2004).
