# Supplementary material for: Nuclear lamin A/C harnesses the perinuclear apical actin cables to protect nuclear morphology
Source: Nat Commun. 2017 Dec 14;8:2123. doi: 10.1038/s41467-017-02217-5 (PMC5730574; doi:10.1038/s41467-017-02217-5)
Supplement: Supplementary file 3 — Description of Additional Supplementary Files [file 41467_2017_2217_MOESM3_ESM.pdf]

Description of Additional Supplementary Files

File Name: Supplementary Movie 1

Description: Dynamic changes in cell orientation and cytoskeletal reorganization under uniaxial cyclic stretching of the substrate. EGFP-LifeAct-transfected mouse embryonic fibroblasts seeded onto the PDMS thin film were re-oriented in response to the substrate stretching (8% strain ratio and 1Hz frequency) by the reorganization of actin cytoskeleton perpendicular to the substrate stretching direction. Each frame was collected every 2 min for 1 h.
