# Supplementary material for: Nuclear lamin A/C harnesses the perinuclear apical actin cables to protect nuclear morphology
Source: Nat Commun. 2017 Dec 14;8:2123. doi: 10.1038/s41467-017-02217-5 (PMC5730574; doi:10.1038/s41467-017-02217-5)
Supplement: Supplementary file 2 — Peer Review File [file 41467_2017_2217_MOESM2_ESM.pdf]

Editorial Note: this manuscript has been previously reviewed at another journal that is not operating a transparent peer review scheme. This document only contains reviewer comments and rebuttal letters for versions considered at Nature Communications.

REVIEWERS' COMMENTS:

Reviewer #1 (Remarks to the Author):

Editorial Note: this Reviewer provided no further comments to the authors.
